# Ankle-Brachial Index as a Predictor of Mortality in Hemodialysis: A 5-Year Cohort Study

**DOI:** 10.5935/abc.20170026

**Published:** 2017-03

**Authors:** Jair Baptista Miguel, Jorge Paulo Strogoff de Matos, Jocemir Ronaldo Lugon

**Affiliations:** Universidade Federal Fluminense (UFF), Rio de Janeiro, RJ - Brazil.

**Keywords:** Ankle Brachial Index / mortality, Measures, Renal Dialysis, Renal Insufficiency, Chronic, Arterial Pressure, Cohort Studies

## Abstract

**Background:**

Abnormal ankle-brachial index (ABI) has been found to be a strong predictor of mortality in some hemodialysis populations in studies with relatively short periods of follow-up, lower than 2 years.

**Objective:**

This study aimed to assess the predictive value of abnormal ABI as a risk factor for death among patients on maintenance hemodialysis after a 5-year follow-up.

**Methods:**

A total of 478 patients on hemodialysis for at least 12 months were included in the study. ABI measurement was performed using a mercury column sphygmomanometer and portable Doppler. Patients were divided into 3 groups according to ABI (low: <0.9; normal: 0.9 to 1.3; and high: >1.3) and followed for a 60-month period.

**Results:**

The prevalence rates of low, normal and high ABI were 26.8%, 64.6% and 8.6%, respectively. The 5-year survival rate was lower in the groups with low ABI (44.1%, P<0.0001) and high ABI (60.8%, P= 0.025) than in the group with normal ABI (71.7%). Cox regression was used to evaluate the association between ABI and mortality, adjusting for potential confounders. Using normal ABI as reference, a low, but not a high ABI was found to be an independent risk factor for all-cause mortality (HR2.57; 95% CI, 1.84-3.57 and HR 1.62; 95% CI, 0.93-2.83, respectively).

**Conclusions:**

long-term survival rates of patients with either low or high ABI were lower than the one from those with normal ABI. However, after adjustment for potential confounders, only low ABI persisted as an independent risk factor for all-cause mortality among hemodialysis patients.

## Introduction

The mortality rate among end-stage renal disease (ESRD) patients is still high and cardiovascular diseases (CVD) are responsible for approximately 50% of the deaths.^1-4^ In addition to ischemic heart disease and cerebrovascular disease, peripheral arterial disease (PAD) is highly prevalent among dialysis patients and its presence is associated with high morbidity and mortality.^5-8^ The ankle-brachial index (ABI) is a simple, inexpensive and non-invasive test^7-9^ that has been shown to have a high sensitivity and specificity for the diagnosis of PAD when compared to angiography, the gold-standard diagnosis method.^10^ This index is based on the fact that systolic blood pressure in the legs is usually equal to or slightly higher than in the upper limbs in healthy individuals. In the presence of artery stenosis, a reduction in pressure occurs distally to the lesion.^11^ In addition, low ABI has a strong correlation with arterial disease in other sites and has been found to be a good predictor of mortality in the general population.^12,13^ Moreover, both low and high ABI have been found to be strong predictors of death among hemodialysis patients.^6-9^

Considering that the usefulness of ABI has already been demonstrated in some hemodialysis populations with a mean follow-up lower than 2 years,^7,8^ the present study aimed to assess the predictive value of ABI as an independent risk factor for death among patients on maintenance hemodialysis after a 5-year follow-up.

## Methods

This is an observational prospective study, with a 5-year follow-up period, performed at six dialysis facilities in Rio de Janeiro State, Brazil. All patients aged 18 to 75 years, who had been on hemodialysis for at least 12 months, were eligible to be included in the study. Written informed consent was obtained and approved, as well the protocol of study, by the local ethical committee, number CEP 23/06. Patients with cancer, anti-HIV positive test, atrial fibrillation, bilateral lower-limb amputation, or dementia and those who refused to participate were excluded from the study. The ABI measurements were taken between March 2006 and September 2007.

### The ankle-brachial index

ABI, defined as the ratio of ankle-to-arm systolic blood pressure, was measured once, at the entrance of the patients in the study, before hemodialysis session and after 5 minutes in supine position. In lower limbs, tibial posterior arteries were used, since the *dorsalis pedis* artery is congenitally absent in 4 to 12% of the population.^14^ Systolic blood pressure was measured twice at each site, in rapid and alternate succession, to obtain a mean value. Standard blood pressure arm cuffs connected to a mercury column were applied to the arm and to each ankle (with the lower end of the bladder just above the malleoli). Ultrasound gel was applied, and a Doppler stethoscope (10 MHz, Super Dupplex, Huntleigh Technology Inc., Manalapan NJ, USA) was used to assess systolic blood pressure. Systolic blood pressure in the upper limb was measured on the brachial artery of the arm contralateral to the vascular access. To calculate ABI, the lowest mean from the ankles was divided by the mean in the arm. All ABI measurements were performed by 3 trained observers (one physician and two medical students) based on the information that inter- and intra-observer variability for Doppler blood pressure measurement is negligible.^5,15^

To evaluate the relationship between ABI and demographics, clinical and laboratory data, the population was divided into three groups according to ABI values: low ABI group (< 0.9), normal ABI group (0.9 to 1.3) and high ABI group (> 1.3).

### Demographic, clinical and laboratory data

Demographics and clinical data were derived from both a structured clinical interview and a database used in all six dialysis facilities. These data included gender, age, race, time on dialysis, primary kidney disease, vascular access, and current smoking status. Comorbidities were defined as following: diabetes; hypertension (pre-dialysis systolic blood pressure ≥ 140 mmHg and/or diastolic pressure ≥ 90 mmHg and/or use of anti-hypertensive drugs); coronary artery disease (exertional angina, current use of coronary vasodilator, past myocardial infarction, and coronary artery bypass graft or percutaneous coronary intervention); stroke sequelae; PAD (current use of peripheral vasodilator, past lower limb artery bypass surgery, angioplasty or non-traumatic lower limb amputation); and hepatitis C seropositivity. Levels of C-reactive protein (CRP) were determined by ultra-sensitive immunoturbidimetric assay, specifically for the study, and the values shown were determined by the time ABI was measured. The remaining laboratory data - basal levels of hemoglobin, serum creatinine, blood urea nitrogen (BUN), equilibrated urea Kt/V (eKt/V), and albumin - were extracted from patients' medical records. To better estimate the impact of bone mineral disturbances on our findings, cumulative exposure was assessed through calculation of the mean of all values for serum calcium, phosphorus and intact parathyroid hormone (i-PTH) measurements along the 36-month period preceding the ABI evaluation or since hemodialysis initiation for patients on dialysis for less than 3 years, as described previously.^16^ Calcium and phosphorus serum levels were measured on a monthly basis and i-PTH every six months. All routine blood analyses were performed in a central laboratory.

### Statistical analysis

Continuous variables were expressed as mean ± SD if distribution was normal and as median and range in case of non-Gaussian distribution. Categorical variables were presented as frequency. Comparison of the means between the ABI groups were performed using analysis of variance (ANOVA) complemented by Bonferroni test or the nonparametric test of Kruskal-Wallis complemented by Dunn test as appropriated. Frequencies were compared by Fisher's exact test. The Kaplan-Meier test was used for analysis of survival, and comparison between curves was made by the Log-Rank test.

Based on a previous pilot study, we estimated that the prevalence of low, normal and high ABI would be approximately 30%, 60% and 10%, respectively. Our study was designed to have a statistical power of 0.8 to detect a difference in survival rate between low and normal ABI of 30%, with a two-sided alpha level of 5%. Thus, after accounting for an expected 20% of drop-out for reasons other than death, the minimal number of participants was estimated to be 450.

Associations of ABI group (low, normal and high) with death risk were analyzed by Cox-regression models: a non-adjusted model, which only included the variable of primary interest, ABI; a model adjusted for demographics and clinical data (gender, age, race, diabetes, time on dialysis, smoking, coronary disease, stroke sequelae) - "Model 1"; and finally, a model in which laboratory variables (serum albumin, hemoglobin, i-PTH, ionized calcium, phosphorus, eKt/V, and CRP) were also included as potential confounders - "Model 2".

The null hypothesis was rejected when P value was < 0.05. The software SPSS, version 18.0 (Chicago, Illinois, USA) was used for the statistical analysis.

## Results

Of a total of 1,170 patients on maintenance hemodialysis in six dialysis facilities, 478 patients were enrolled in the study. Demographic and laboratory characteristics of patients are listed in Table 1. Median age was 54 (18 -75) years, 56% were males, and 14.9% and 50.6% had diabetes and hypertension as the primary kidney disease, respectively. Median time on dialysis was 59 (12 - 427) months, and longer than 3 years for 73% of patients.

**Table 1:** Demographic and laboratory characteristics of the population (n = 478)

Male gender, (%)	268 (56%)
Age (years)	54 (18-75)
Race	
White	221 (46.2%)
Non-White	257 (53.8%)
Time on dialysis (months)	59 (12-427)
Primary kidney disease, (%)	
Diabetic nephropathy	71 (14.9%)
Hypertensive nephrosclerosis	242 (50.6%)
Chronic glomerulonephritis	41 (8.6%)
Unknown	62 (13.0%)
Polycystic kidney disease	21 (4.4%)
Lupus nephropathy	8 (1.7%)
Others	33 (6.9%)
Comorbidities, (%)	
Diabetes	81 (16.9%)
Hypertension	291 (60.9%)
Smoking	73 (15.3%)
Coronary artery disease	114 (23.9%)
Stroke sequelae	16 (3.3%)
Peripheral artery disease	86 (18%)
Patients < 3 years of hemodialysis, (%)	
Parathyroidectomy	29 (6.1%)
Anti-HCV positive test, (%)	101 (21.1%)
HB HBsAg positive test	13 (2.7%)
eKt/V	1,51 ± 0,40
Hemoglobin (g/dL)	11,4 ± 1,6
Albumin (g/dL)	3,8 ± 0,3
Calcium (mg/dL)	4,6 ± 0,3
Phosphorus (mg/dL)	5,4 ± 1,2
i-PTH (pg/mL)	370 (10-2,500)
CRP (mg/L)	4,7 (0,1 - 150)

CRP: C-reactive protein; eKt/V: equilibrated Kt/V; HCV: hepatitis C virus; i- PTH :Intact parathyroid hormone; HBsAg: Hepatitis B Surface Antigens; Values are expressed as median (range), mean ± SD or by frequency.

The prevalence of low, normal and high ABI was 26.8%, 64.6% and 8.6%, respectively. Table 2 shows the characteristics of each ABI group. Male gender prevailed in the high ABI group, when compared to the low and normal ones. Patients in the low ABI group were significantly older than those in the normal and high ABI groups. The prevalence of diabetes, PAD and non-traumatic amputation was significantly lower in the normal ABI group, when compared to both low and high ABI groups. Coronary artery disease and stroke sequelae were more frequent in the low ABI group than in the normal ABI group. No difference was found regarding arm blood pressure measurements between the groups.

**Table 2:** Demographics according to ankle-brachial index (ABI) classification

Variables	ABI		
	Low (n=128)	Normal (n=309)	High (n=41)
**Male (%)**	53.1	53.7	80.5*
Age (years)	62 (20 to 77)	49 (18 to 75)**	54 (27 to 71)**
Race (White), %	45	44	61^†^
Time on dialysis (months)	57 (13 to 321)	59 (12 to 292)	65 (13 to 427)
Primary kidney disease, f (%)			
Diabetes	25.0	8.4**	31.7^†^
Hypertension	51.6	52.1	36.6
Chronic glomerulonephritis	3.9	11.0**	4.9
Polycystic kidney disease	3.9	4.9	2.4
Lupus nephropathy	-	2.3	2.4
Others	8.6	6.5	4.9
Unknown	7.0	14.9	17.1
**Comorbidities (%)**			
Diabetes	30.5	9.4**	31.7^†^
Hypertension	65.6	60.5	48.8
Smoking	17.2	15.2	9.8
Coronary artery disease	25.0	15.2^#^	12.2
Stroke sequelae	8.6	1.6**	-
Peripheral artery disease	27.3	7.4**	24.4^†^
Nontraumatic amputation	7.8	1.3**	9.8^†^
Parathyroidectomy	4.7	6.5	7.3
Positive HBsAg	4.7	2.3	-
Positive anti-HCV test (%)	20.3	19.4	36.6^††^

HCV: hepatitis C virus; HBsAg: Hepatitis B Surface Antigens; Values are expressed by frequency and median (range);

* p < 0.01 vs. low and normal ABI;

† P < 0.01 vs. normal ABI;

# p < 0.05 vs. low ABI;

** p < 0.01 vs. low ABI;

†† p < 0.05 vs. normal ABI.

Laboratory findings of each group are shown in Table 3. The low ABI group had higher CRP and lower serum albumin than the normal ABI group. Serum creatinine was lower in the low ABI group than in normal and high ABI groups. The high ABI group had increased serum phosphorus levels and calcium × phosphorus product, when compared to the normal and low ABI groups. The high ABI group also had increased i-PTH levels compared to the low ABI group.

**Table 3:** Laboratory findings according to ABI classification

Parameters	ABI		
	Low (n=128)	Normal (n=309)	High (n=41)
CRP (mg/L)	6.4 (0.2-150)	3.9 (0.1-150)*	4.3 (0.2-41)
Albumin (g/dL)	3.74 ± 0.31	3.84 ± 0.30*	3.72 ± 0.36
BUN (mg/dL)	69 ± 22	68 ± 22	76 ± 22
Creatinine (mg/dL)	10.6 ± 2.8	11.9 ± 3.0*	12.2 ± 2.8*
eKt/V	1.51 ± 0.41	1.53 ± 0.42	1.36 ± 0.23^†^
Hemoglobin (g/dL)	11.6 ± 1.6	11.2 ± 1.7	12.2 ± 2.8
i-PTH (pg/mL)	297 (28 - 2,202)	386 (4 - 2,500)	489 (10 - 2,160)**
Ionic calcium (mg/dL)	4.6 ± 0.3	4.6 ± 0.3	4.6 ± 0.4
Phosphorus (mg/dL)	5.3 ± 1.2	5.4 ± 1.1	5.8 ± 1.4^††^
Ca x P product (mg^2^/dL^2^)	24.1 ± 5.7	24.7 ± 5.5	27.1 ± 6.3^††^

CRP: C-reactive protein; BUN: Blood urea nitrogen; eKt/V: Equilibrated Kt/V; i-PTH: Intact parathyroid hormone; Values are expressed by the median (limits) or by the mean ± SD;

* p < 0.01 vs. low ABI;

† p < 0.05 vs. normal ABI;

** p < 0.05 vs. low ABI;

†† p < 0.05 vs. low and normal ABI.

**Figure 1:**
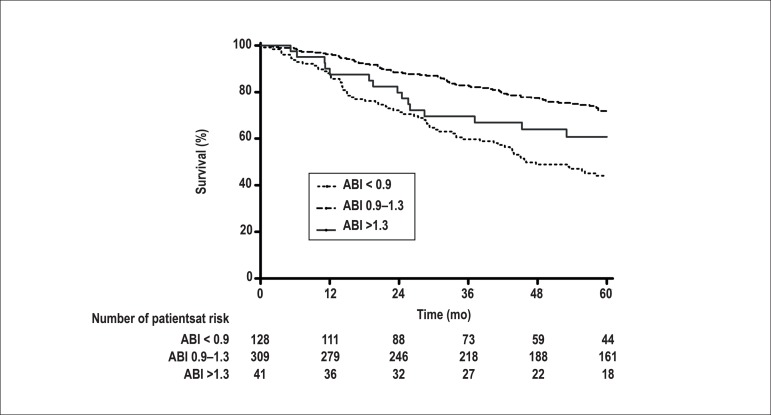
Survival curves for the first 5 years of follow-up according to ankle-brachial index ( ABI) at baseline

After a 5-year follow-up period, 158 of 478 patients died, 69 lost their follow-up due to a change of dialysis facility and 28 underwent kidney transplantation. The survival curves according to the ABI group are presented in Figure 1. When the 5-year survival rates were compared, values were lower in the groups of altered ABI (44.1% for low ABI and 60.8% for high ABI) than in the normal ABI group (78%), P <0.0001 and P = 0.025, respectively.

The association of ABI with mortality risk in the Cox proportional hazard models is shown in Table 4. In the non-adjusted model, low ABI was associated with increased mortality risk (HR 2.57, 95%CI1.84-3.57), but the association of high ABI with death (HR 1.62, 95%CI0.93-2.83) was not significant. In the multivariate analysis, after adjustment for demographics and comorbidities (Model 1), low ABI persisted significantly associated with all-cause mortality (HR1.83, 95%CI1.28-2.63), accompanied by age (HR1.02, 95%CI1.01-1.04). After further adjustment for laboratory variables (Model 2), low ABI (HR 1.69, 95%CI 1.14-2.51) and age (HR 1.02[per year], 95%CI 1.01-1.04) again persisted as significantly associated with all-cause mortality. Here, stroke sequelae (HR 2.25, 95%CI 1.09-4.67) and CRP (HR 1.02[per mg/L], 95%CI 1.01-1.03) also were found to be significantly associated with increased mortality risk.

**Table 4:** Predictors for overall mortality using Cox proportional hazards models

Variables	Non-adjusted	Model 1*	Model 2**
	HR (95%CI)	HR (95%CI)	HR (95%CI)
**ABI**			
Normal (ref.)	1.00	1.00	1.00
High	1.62 (0.93-2.83)	1.47 (0.83-2.60)	1.16 (0.60-2.26)
Low	2.57 (1.84-3.57)	1.83 (1.28-2.63)	1.69 (1.14-2.51)
Gender (male)	-	1.23 (0.89-1.71)	1.25(0.86-1.81)
Age (years)	-	1.02 (1.01-1.04)	1.02 (1.01-1.04)
Race (White)	-	0.98 (0.71-1.36)	0.95 (0.66-1.37)
Diabetes (y/n)	-	1.37 (0.93-2.03)	1.37 (0.88-2.13)
Time on dialysis (mo)	-	1.00 (0.98-1.00)	1.00 (0.99-1.00)
Smoking (y/n)	-	1.23 (0.83-1.82)	1.27 (0.84-1.92)
Coronary disease (y/n)	-	1.13 (0.77-1.67)	1.06 (0.69-1.63)
Stroke Sequelae (y/n)	-	1.73 (0.89-3.39)	2.25 (1.09-4.67)
**Laboratory parameters**			
Albumin (g/dL)	-	-	0.82 (0.44-1.52)
Hemoglobin (g/dL)	-	-	0.97 (0.87-1.09)
i-PTH (pg/mL)	-	-	1.00 (0.99-1.00)
Calcium (mg/dL)	-	-	1.06 (0.60-1.89)
Phosphorus (mg/dL)	-	-	0.93 (0.78-1,10)
eKt/V	-	-	0.85 (0.53-1.36)
CRP (mg/L)	-	-	1.02 (1.01-1.03)

Values expressed as hazard ratios (HR) and 95% confidence interval (CI);

* Adjusted for demographics date and comorbidities;

** Adjusted for demographics data, comorbidities and laboratory parameters; PTH -Intact parathyroid hormone; eKt/V -equilibrated Kt/V; CRP - C reactive protein.

## Discussion

ABI is an easy, reliable and non-invasive test, that has been used as a diagnostic tool for PAD, a condition highly prevalent among hemodialysis patients.^5,8^ It has also been shown to be a useful marker of diffuse atherosclerotic disease as well as a predictor of mortality in patients on hemodialysis and in general population.^5,7,8,12,13^ The relationship between ABI and CVD has also been demonstrated by a negative correlation between ABI and intimal medial thickness,^17^ and by an inverse correlation between ABI and left ventricular mass in hypertensive patients without clinical manifestations of PAD.^18^

The survival curves were significantly different between the ABI groups in the current study. Survival was lower in both low and high ABI groups when compared to the normal one. These findings point to the importance usefulness of ABI as an important predictor of mortality in hemodialysis population. Low ABI was found to be associated with higher mortality rate in general population^12-15^ as well as in patients with chronic kidney disease, stages 3 to 5^19^ and hemodialysis patients.^7,8,20^ High ABI has also been associated with increased mortality in studies involving hemodialysis patients.^8,20^

Abnormal values of ABI as predictors of death were assessed by Cox proportional hazards models. In the non-adjusted model, in which only three bands of ABI were taken into account, and the normal ABI band was taken as reference, only low ABI was associated with a significant death risk. The multivariate analysis was performed in two steps. Firstly, we developed the Model 1, in which the association of ABI bands with mortality was adjusted for gender, age, race, diabetes status, time on dialysis, smoking, coronary disease, and stroke sequelae. On a second step, in Model 2, laboratory parameters were also added as potential confounding factors. Our findings showed that low ABI persisted as an independent risk factor for all-cause mortality even after adjustment for all demographics, comorbidities and laboratory variables. On the other hand, we found that high ABI did not represent an independent risk factor for all-cause mortality. This is in disagreement with previous studies,^8,20^ but the small sample size in our analysis could have reduced the chance of detecting a true effect of high ABI due to the low statistical power.

Also interesting was the finding that diabetes, *per se,* did not represent an independent determinant for mortality. This finding is consistent with previous studies^21,22^ suggesting that only diabetic patients on hemodialysis having arterial disease present a greater risk of death. Moreover, diabetes was not a risk factor for death in hemodialysis when patients with PAD were excluded from the sample.^23^

Age, baseline CRP levels and stroke sequelae were confirmed as independent risk factors for death during the 5-year follow-up period. The first two variables are well-known risk factors for death in hemodialysis^24,25^, confirming the association between a single baseline CRP measurement and long-term mortality risk. Stroke sequelae may represent the association between low ABI and diffuse atherosclerotic disease, and could be seen as a link between low ABI and high mortality rate in hemodialysis patients.

Among 478 patients enrolled, the frequency of normal, low and high ABI was 64.6%, 26.8% and 8.6%, respectively. There was a predominance of males among patients with high ABI. Patients in the low ABI group were older than those in the other two groups. We found a higher prevalence of diabetes in both low and high ABI groups, in comparison to the group with normal ABI. The high prevalence of diabetes among low ABI patients could be attributed to the presence of macrovascular disease, whereas the predominance of diabetes in the group of high ABI could be explained by the greater prevalence of vascular calcification in diabetic patients.^8^ Vascular calcification can cause arterial stiffness, and consequently increased ABI.

Regarding hypertension, we did not detect significant differences between the ABI groups. We also found no association between smoking and the risk of abnormal ABI. Perhaps, the low prevalence of smoking in our population could have mitigated such effect. Moreover, the absence of such correlation could be attributed to data collection strategy, since we considered only current smokers in our study. The association between smoking and PAD in hemodialysis patients was controversial in previous studies.^6,8,20,26^

The low ABI group showed a higher prevalence of coronary artery disease, stroke sequelae, PAD and non-traumatic amputation when compared to the normal ABI group. The association between low ABI and generalized atherosclerotic disease was also found in previous studies.^5,6,8,27^ It should be underscored that the high ABI group also presented a higher prevalence of PAD and non-traumatic amputation in relation to the normal group.

The positive correlation between atherosclerosis and inflammation, demonstrated in previous studies in both general population and hemodialysis patients^28,29^ could also be observed in our study, considering the variables CRP and serum albumin. The group of low ABI showed higher levels of CRP and lower serum albumin than the normal group. This finding is also consistent with studies evaluating specifically PAD in both general population and hemodialysis patients.^30-32^ The lower serum creatinine levels in the low ABI group suggest that some degree of malnutrition was present in these patients, a comorbidity correlated with inflammation.

Ionized calcium, phosphorus and i-PTH levels were used to evaluate bone and mineral disturbances. The levels of ionized calcium were similar in the three groups, whereas phosphorus levels and the calcium × phosphorus product were higher in the high ABI group than in the other two groups reflecting, probably, a putative role of phosphorus in vascular calcification. These results are in agreement with a prior study, in which the association between serum phosphorus and Ca x P levels were observed only in patients with ABI >1.4 or incompressible ankle arteries.^33^

It should be stressed that, differently from other studies, we did not perform a merely cross-sectional analysis of the association between current ionized calcium and phosphorus levels and the presence of PAD. In fact, in our study, calcium and phosphorus data represent the mean of values of monthly measurements of these variables during a long period of up to 36 months preceding ABI evaluation. Thus, our data point against a direct association between hypercalcemia or hyperphosphatemia and low ABI.

i-PTH values were higher in the group with ABI >1.3 than in the group with ABI <0.9. A negative association of i-PTH levels with PAD, as well as with cardiac and aortic valve calcification was also found in previous studies.^27,34^ The reasons for this negative association are not clear but might be related to a tendency toward a soft tissue calcification in low-turnover bone disease or to the association between low i-PTH levels and malnutrition.^35^ It is worth pointing out, however, that the inverse association between i-PTH levels and the presence of PAD is not a uniform finding. 

Considering the high prevalence of PAD, its consequences and the current lack of effective therapies for hemodialysis patients, we think that the routine measurement of ABI could identify patients in higher long-term risk of death, who could benefit from early detection of PAD and interventions on risk factors associated with low ABI, such as inflammation, in attempt to change the apparently inexorable course of this disease.

This study has some limitations that deserve consideration. Several established risk factors for PAD in general population, as smoking, could not be properly evaluated, since the data collection considered only the current state and not the total burden of exposition to it. We also could not distinguish between overall and cardiovascular cause of mortality due to the lack of accurate information. Another limitation is that the studied population could not be representative of the national one a nationwide feature, since all patients are from Rio de Janeiro State. On the other side, the strengths of our study include the assessment of ABI by Doppler, the gold-standard method, its prospective design and the long follow-up period. Most of similar studies observed patients for no more than 2 years. There is no standardized definition for "long-term" concerning the follow-up in clinical research, but its meaning can be viewed as dependent on the disease, treatment and populations studied.^36^ Considering a mean annual mortality rate of 15% to 20% in hemodialysis population, it seems reasonable to label a period of 5 years in our population as a long-term follow-up.

## Conclusions

Our findings showed a high frequency of abnormal ABI among patients in hemodialysis. Long-term survival rates of patients with either low or high ABI were lower than the one from those with normal ABI. However, after adjustment for potential confounders, only low ABI persisted as an independent risk factor for all-cause mortality among hemodialysis patients. In addition, the relatively higher risk of death for diabetic patients was reversed after adjustment for ABI.
